# Relationship between children’s autism spectrum disorder and parental anxiety and burnout

**DOI:** 10.12669/pjms.41.2.9979

**Published:** 2025-02

**Authors:** Mahrukh Akram, Syed Muhammad Zulfiqar Hyder Naqvi, Nazia Jameel

**Affiliations:** 1 Mahrukh Akram, MPH Department of Community Medicine, Dow International Medical College, Gulzar-e-Hijri Gulshan-e-Iqbal; 2 Syed Muhammad Zulfiqar Hyder Naqvi, MSc Epidemiology and Biostatistics Department of Community Medicine, Baqai Medical College, 51 Deh-Tor, Gadap Road, Karachi, Pakistan; 3 Nazia Jameel, MPH Department of Community Medicine, Baqai Medical College, 51 Deh-Tor, Gadap Road, Karachi, Pakistan

**Keywords:** Child, Autism Spectrum Disorder, Parents, Anxiety, Burnout, Psychological

## Abstract

**Objective:**

To determine the association between children’s autism spectrum disorder with parental anxiety and burnout in Karachi, Pakistan.

**Methods:**

A cross-sectional study was conducted at Baqai Institute of Health Sciences, Baqai Medical University from December 2022, to November, 2023. Data collection was performed at three rehabilitation centers for autistic children in Karachi. The study population consisted of parents of autistic children. GAD-7 and Parental Burnout Assessment scales were used to assess the anxiety and burnout level among parents. Data were analyzed using SPSS version 21.

**Results:**

The study results showed that 49 (24.5%) parents had moderate anxiety while 38 (19.0%) had severe anxiety. Besides, 36 (18.0%) of them were at risk of burnout while 8 (4.0%) were diagnosed with burnout. Furthermore, gender of child (p=0.01), father’s age (p=0.015) and type of family (p=0.001) were significantly associated with the anxiety level whereas father’s age (p=0.036), father’s education (p=0.006) and type of family (p=0.046) were significantly associated with the burnout level of the parents.

**Conclusion:**

This study results showed significant association of certain child and parental characteristics such as child’s gender, father’s age and type of family with parental anxiety level and of father’s age, father’s education and type of family with parental burnout.

## INTRODUCTION

Autism Spectrum Disorder (ASD) is characterized by disturbances in three behavioral domains which includes social interaction, communication, and repetitive behaviors.^1^ The early symptoms in an autistic child are poor eye contact and difficulty in social interactions.^2^ ASD diagnosis is based on clinical observations as there are no known biomarkers that can detect or predict Autism.^2^ The prevalence of ASD is rapidly increasing but its cause is still unknown,^3^ though it has been reported to be more common in males than in females.^4^

The World Health Organization has reported that 1 in 100 children have ASD.^5^ According to the Autism Society of Pakistan, it has been estimated that there are around 350,000 children diagnosed with autism in Pakistan.^6^ Moreover, many cases of autism remain undiagnosed due to lack of awareness and understanding among parents therefore the actual figures of ASD may be even higher.

Parents of children diagnosed with ASD have to face several challenges which include giving increased attention, time and financial expenses.^7^ Diagnosis of ASD affects the dynamics of family and past studies have shown that higher level of burnout is found in parents of autistic children as compared to the parents of healthy children. The demanding role of being a parent of an autistic child, difficulty to get a proper diagnosis and scarcity of relevant information related to the therapies and rehabilitation, minimum social support and difficulties in participating in social life are the major contributing factors. Anxiety is defined by the American Psychological Association as “an emotion characterized by intense feelings of tension, worried thoughts, and physical changes like increased blood pressure”.^8^ Parental Burnout is defined as a condition characterized by intense exhaustion related to the role of parenting, emotional distancing from one’s own children, and a lack of fulfillment as a parent.^9^

Literature shows that parents of autistic children suffer from increased level of anxiety and have greater risk of psychological disturbances.^10^,^11^ To the best of authors’ knowledge though, local evidence on the effect of ASD on parent’s mental well-being is limited at best.^7^,^12^,^13^ It is important to investigate the impact of this disorder on parents in this region so that effective measures can be taken and policies can be made to extend the social support for such families in order to improve their mental well-being. This study was therefore conducted to determine the relationship between child’s ASD and parent’s anxiety and burnout in Karachi, Pakistan.

## METHODS

A cross-sectional study was conducted at Baqai Institute of Health Sciences, Medical University from December 2022, to November, 2023. Data collection was performed at three rehabilitation centers for autistic children in Karachi. Parents who have at least one child diagnosed with autism spectrum disorder without any known comorbids like epilepsy, attention deficit hyperactivity disorder or intellectual disability were included in this study whereas parents of children with physical and mental disabilities other than autism spectrum disorder or with any diagnosed chronic medical conditions were excluded from the study. Keeping the percentage frequency of the study outcome at 50% for most liberal estimate, 95% confidence level and 7% precision the minimum required sample size was calculated to be 196 participants by using the following formula: n = z^2^(p)(1-p)/c^2^. Non probability purposive sampling technique was used to include participants in the study. Each parent was approached and given explanation about the study. Written informed consent was obtained from each participant and a copy of the consent form was given to them. Data were then collected by interviewing the parents of autistic children using the study questionnaire. The interview took a total of 10 minutes.

### Ethical Approval:

Approval of study was taken from Baqai Institute of Health Sciences (Reference # FHM 96-2022) dated 12^th^ December, 2022. Permission was also obtained from other institutions from where the data were collected.

The study questionnaire consisted of three sections; section-A for demographic information, section-B for Parental Burnout Assessment Scale, and section-C for Generalized Anxiety Disorder-7 Scale. Section-A contained 11 questions about demographic profile of the autistic children and their parents. Section-B contained Parental Burnout Assessment Scale (PBA). PBA is a 23-item tool that examines four factors: exhaustion, emotional distancing, saturation and contrast. It has been previously validated to measure parental burnout.^14^,^15^ The total parental burnout assessment score is calculated by adding the score obtained on all items (minimum score of 0 and maximum score is 138). Parental burnout is considered higher if a higher score is obtained. Diagnosis of parental burnout is labeled from a score of 86 whereas risk of parental burnout is labeled from a score of 53. Section-C contained the Generalized Anxiety Disorder (GAD-7) scale. GAD-7 consists of seven items that measures worry and anxiety symptoms. Each item is scored on a four point Likert scale (0-3) with total scores ranging from 0-21 with a higher scores reflecting greater severity of anxiety. The validity of GAD-7 has been assessed for general population previously.^16^,^17^ Scoring of GAD-7 scale is done as scores of 5, 10, and 15 are taken as the cut-off points for mild, moderate and severe anxiety, respectively.

### Statistical Analysis:

Data were entered and analyzed by SPSS version 21. Descriptive analysis such as frequencies and percentages were executed for categorical variables while means and standard deviation were calculated for continuous variable. Inferential analysis was performed by using chi square test for checking associations between demographic characteristics of the children and parents and the study outcomes. The significance level was set at 0.05.

## RESULTS

Against the calculated sample size of 196, a total of 200 respondents were included in the study with a response rate of 89.3%. The mean age of the study children was 6.87±3.55 years, 93 (46.5%) of them were 6-9 years old, 137 (68.5%) were male, 160 (80%) of the parents were females, the mean age of fathers was 40.7±7.53 years, 108 (54.0%) of them were aged above 40 years, the mean age of mothers was 35.89±6.84 years, 144 (72.0%) of them were aged less than 40 years age, 139 (69.5%) fathers were either graduate or post graduate, 117 (58.5%) of the mothers were either graduate or post graduate, 102 (51.0%) of lived in nuclear family setup, 81 (40.5%) of parents had monthly household income less than 50,000 rupees whereas 74 (37.0%) had monthly household income from 51,000 to 100,000 rupees, 161 (80.5%) of the children had either mother or father working, 140 (70.0%) of the parents didn’t use any medication for their child, 192 (96.0%) of the children received therapy whereas 161 (80.5%) of them received more than one type of therapy.

**Fig.1:**
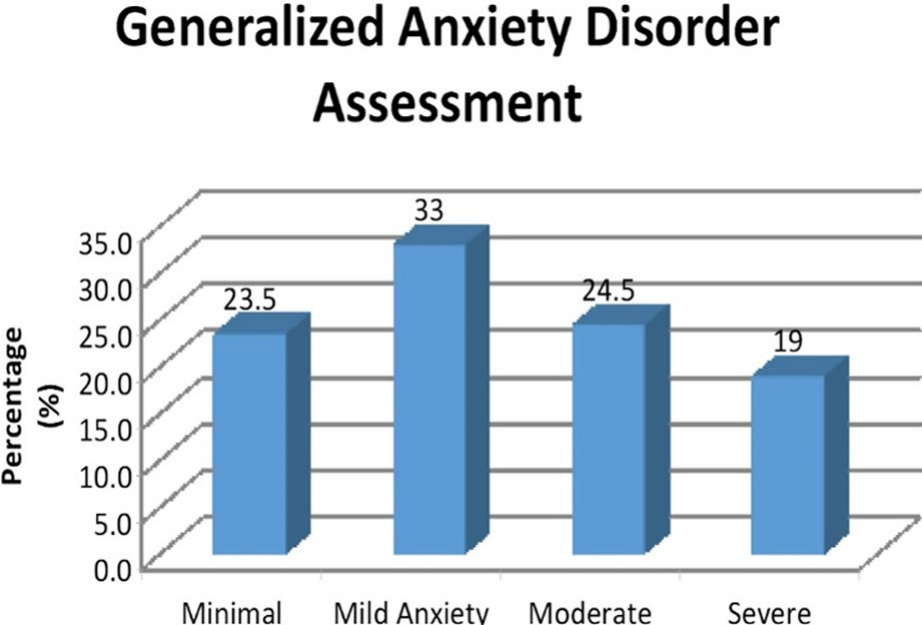
Generalized Anxiety Disorder Assessment. The assessment of parental anxiety is divided into four categories, namely Minimal, Mild, Moderate, and Severe.

Furthermore, 24.5% (n=49) parents were suffering from moderate anxiety whereas 19.0% (n=38) had severe anxiety (Fig.1). Moreover, gender of child (p=0.010), age of father (p=0.015) and type of family (p=0.001) were significantly associated with the anxiety level of the parents where parents of male child were more likely to have moderate and severe anxiety as compared to the parents of female child; fathers who were aged less than 40 years were more likely to have moderate and severe anxiety as compared to those who were aged 40 years or above, and parents who lived in joint families were more likely to have severe anxiety as compared to those who lived in nuclear families (Table-I).

**Table-I:** Association between Participant Characteristics and Anxiety Level of Parents.

Participant Characteristics (n=200)	Anxiety Level				p

	Minimal Anxiety	Mild Anxiety	Moderate Anxiety	Severe Anxiety	

	n=47	n=66	n=49	n=38	

	Count (%)	Count (%)	Count (%)	Count (%)	
Child Age					
Less than 5 years	14 (21.2)	19 (28.8)	20 (30.3)	13 (19.7)	0.707
6 to 9 years	20 (21.5)	34 (36.6)	21 (22.6)	18 (19.4)	
10 years or above	13 (31.7)	13 (31.7)	8 (19.5)	7 (17.1)	
Gender of Child					
Male	26 (19.0)	41 (29.9)	41 (29.9)	29 (21.2)	0.010
Female	21 (33.3)	25 (39.7)	8 (12.7)	9 (14.3)	
Gender of Parent					
Male	14 (35.9)	12 (30.8)	7 (17.9)	6 (15.4)	0.219
Female	33 (20.5)	54 (33.5)	42 (26.1)	32 (19.9)	
Mother Age					
Less than 40 years	30 (20.8)	46 (31.9)	37 (25.7)	31 (21.5)	0.289
40 years or above	17 (30.4)	20 (35.7)	12 (21.4)	7 (12.5)	
Father Age					
Less than 40 years	17 (18.5)	24 (26.1)	27 (29.3)	24 (26.1)	0.015
40 years or above	30 (27.8)	42 (38.9)	22 (20.4)	14 (13.0)	
Father Education					
Illiterate/able to read and write/primary/religious education only	4 (18.2)	3 (13.6)	8 (36.4)	7 (31.8)	0.312
Secondary/ intermediate	8 (20.5)	15 (38.5)	9 (23.1)	7 (17.9)	
Graduation/post-graduation	36 (25.2)	48 (34.5)	32 (23.0)	24 (17.3)	
Mother Education					
Illiterate/able to read and write/primary/religious education only	3 (18.8)	4 (25.0)	6 (37.5)	3 (18.8)	0.934
Secondary/ intermediate	15 (22.4)	23 (34.3)	16 (23.9)	13 (19.4)	
Graduation/post-graduation	29 (24.8)	39 (33.3)	27 (23.1)	22 (18.8)	
Does child take any medication?					
Yes	11 (18.3)	27 (45.0)	10 (16.7)	12 (20.0)	0.075
No	36 (25.7)	39 (27.9)	39 (27.9)	26 (19.6)	
Type of Family					
Nuclear	31 (30.4)	36 (35.3)	26 (25.5)	9 (8.8)	0.001
Joint	16 (16.3)	30 (30.6)	23 (23.5)	29 (29.6)	
Monthly Household Income (Rs.)					
Less than 50000	13 (16.0)	27 (33.3)	23 (28.4)	18 (22.2)	0.248
51000 to 100000	18 (24.3)	28 (37.8)	16 (21.6)	12 (16.2)	
More than 100000	16 (35.6)	11 (24.4)	10 (22.2)	8 (17.8)	
Among parents who is working?					
Either Mother or Father	35 (21.7)	53 (32.9)	43 (26.7)	30 (18.6)	0.425
Both Mother and Father	12 (30.8)	13 (33.3)	6 (15.4)	8 (20.5)	

**Fig.2:**
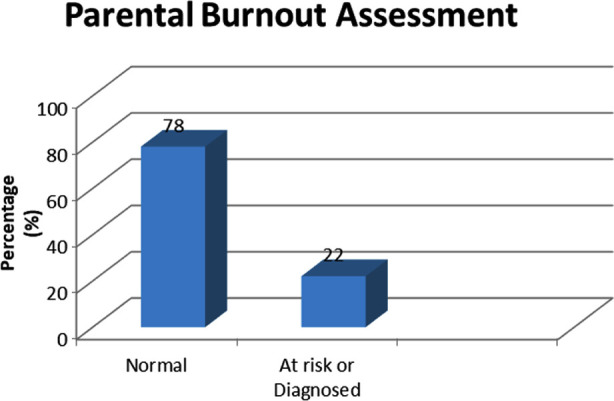
Parental Burnout Assessment. The results of Parental Burnout Assessment were divided into categories, namely, Normal and at risk or Diagnosed.

Moreover, 78% (n=156) parents were normal whereas 22% (n=44) were labeled as diagnosed or at risk of burnout (Fig.2). Furthermore, age of father (p=0.036), education of father (p=0.006) and type of family (p=0.046) were significantly associated with the burnout level of the parents where fathers who were aged less than 40 years were more likely to be at risk or had diagnosis of burnout as compared to those who were aged 40 year or above; fathers who were illiterate/able to read and write/primary/religious education only were more likely to be at risk or burnout as compared to fathers who had secondary/intermediate education or graduate or postgraduate level of education and parents living in joint families were more likely to be at risk or had diagnosis of burnout as compared to the parents living in nuclear families (Table-II).

**Table-II:** Association between Participant Characteristics and Burnout Level of Parents.

Participant Characteristics (n=200)	Burn out level		p

	Normal	At Risk/Diagnosis	

	n=156	n=44	

	Count (%)	Count (%)	
Child age			
Less than 5 years	51 (77.3)	15 (22.7)	0.212
6 to 9 years	69 (74.2)	24 (25.8)	
10 years or above	36 (87.8)	5 (12.2)	
Gender of Child			
Male	107 (78.1)	30 (21.9)	0.548
Female	49 (77.8)	14 (22.2)	
Gender of Parent			
Male	34 (87.2)	5 (12.2)	0.088
Female	122 (75.8)	39 (24.2)	
Mother age			
Less than 40 years	110 (76.4)	34 (23.6)	0.247
40 years or above	46 (82.1)	10 (17.9)	
Father age			
Less than 40 years	66 (71.7)	26 (28.3)	0.036
40 years or above	90 (83.3)	18 (16.7)	
Father Education			
Illiterate/able to read and write/primary/religious education only	12 (54.5)	10 (45.5)	0.006
Secondary/ intermediate	28 (71.8)	11 (28.2)	
Graduation/post-graduation	116 (83.5)	23 (16.5)	
Mother Education			
Illiterate/able to read and write/primary/religious education only	11 (68.8)	5 (31.3)	0.242
Secondary/ intermediate	49 (73.1)	18 (26.9)	
Graduation/post-graduation	96 (82.1)	21 (17.9)	
Does the child take any medication?			
Yes	47 (78.3)	13 (21.7)	0.550
No	109 (77.9)	31 (22.1)	
Type of family			
Nuclear	85 (83.3)	17 (16.7)	0.046
Joint	71 (72.4)	27 (27.6)	
Monthly household income (Rs.)			
Less than 50000	58 (71.6)	23 (28.4)	0.133
51000 to 100000	59 (79.7)	15 (20.3)	
More than 100000	39 (86.7)	6 (13.3)	
Among parents who is working?			
Either Mother or Father	124 (77.0)	37 (23.0)	0.328
Both Mother and Father	32 (82.1)	7 (17.9)	

## DISCUSSION

The study results revealed that 43.5% of the parents had moderate to severe anxiety levels. Likewise, an earlier study reported that 47.8% of the parents of autistic children had moderate to severe anxiety.^18^ Similarly, another study reported that 45.9% of caregivers of autistic children had higher level of anxiety.^19^ In contrast to these findings though, one study reported the prevalence of anxiety to be only 12% in parents of autistic children.^19^ The use of different scales for assessment may have resulted in the difference found in these studies.

In this study, no statistically significant association was found between the child age and the anxiety levels of the parents. Likewise, two earlier studies reported no statistically significant difference in level of parental anxiety among different child ages.^20^,^21^ This could possibly be due to the fact that irrespective of improvement in intellectual and social function with increasing age, caring for such children always remains a challenge for their parents.

Furthermore, younger age of father was significantly associated to moderate and severe anxiety. Unlike the study results though, a study reported that a parent’s or caregiver’s age was not significantly associated with anxiety level.^20^ This may be due to difference in study sample size and difference in assessment scales used to assess anxiety. Also, the study was conducted in a different country so the study population was different.

Moreover, the type of family and the anxiety level of parents were significantly associated. An earlier study reported that parents of autistic children do not receive much acceptance and social support from extended family members and blame is put on mothers for the child behavior.^12^ On the contrary, another study reported that social support in joint family systems protect parents from anxiety.^21^ Further exploration of this finding is therefore recommended by the authors.

Moreover, younger fathers were more likely to have moderate to severe anxiety in our study. An earlier study though reported contrary findings in this regard.^21^ The reason for this may be that the setup of our society is different and younger fathers are less stable financially to raise the family. Their child with special needs is an added pressure on them.

Furthermore, a greater percentage of mothers of autistic children had moderate to severe anxiety as compared to fathers, though these findings were not statistically significant. The findings are consistent with the results reported by an earlier study.^22^ The societal pressure to raise autistic children is greater on mothers than fathers; moreover, the delayed milestones of their children may put added pressure on the mothers hence they suffer from more anxiety. While treating an autistic child, it is very important to evaluate the parents, particularly mothers, for presence of any psychological disturbances.^10^, ^11^

The study results revealed that 22% of the parents of autistic children were at risk or diagnosed with burnout. Due to lack of pertinent local literature a meaningful comparison could not be made, but this percentage is alarmingly high and needs urgent attention for better physical and mental functioning of such parents. Mental health screening of the parents of autistic children is suggested to be made an essential component of the management protocol of such children to ensure a holistic approach for dealing with this illness.

Furthermore, parents of autistic children who lived in joint families were more likely to be at risk of burnout in this study. This finding suggests that the parents living in joint families do not receive increased support which leads to higher burnout levels. On the contrary, an earlier study reported that increase social support leads to increased psychological wellbeing among parents.^23^ In Pakistani population the structure of the family system is different as compared to other countries. Parents living in joint family system have to fulfill the expectations of all the people living in the family system that may be a source of their burnout.^12^

Moreover, mothers of autistic children were more likely to be at risk or diagnosis of burnout than fathers, though the results were not statistically significant. This finding was in line with the published literature.^24^ In Pakistani society mothers bear major responsibilities for the care and upbringing of their children and as a result may become more tired from childcare duties than fathers.

### Strength and Limitations:

Collecting data from multiple sites may be considered strength of the study. With regards to study limitation, it is acknowledged that due to cross-sectional nature of the study, a causal relationship between participants’ characteristics and the study outcomes could not be established.

## CONCLUSION

The results of our study have shown that the many child and parental characteristics such as child’s gender, father’s age and type of family were significantly associated with anxiety level whereas father’s age, education and type of family had significant association with burnout among parents of children with ASD.

Mental health screening of the parents of autistic children is suggested to be made an essential component of the management protocol of such children. Parental counseling services should be prioritized for younger fathers and those with lower education who are more at risk to develop anxiety and burnout. Moreover, parents of male children and those living in joint families should be prioritized for counseling along with efforts to reduce stigma related to autism among masses through advertisements and community awareness camps. Policies should be made to improve the quality of life of such families by social inclusion, financial support programs and greater educational opportunities for their children. In addition, development of a national registry for autistic children is also strongly recommended for prioritizing relevant policy development and implementation.

### Author`s Contribution:

**MA:** Conception and design, acquisition of data; drafting the article.

**SMZHN:** Conception and design, and analysis and interpretation of data; and revising the article critically for important intellectual content

**NJ:** Literature search, critical review.

All authors have approved the final version and are accountable for the integrity of the study.
